# Essential Oil Compositions of *Pinus* Species (*P. contorta* Subsp. *contorta*, *P. ponderosa* var. *ponderosa*, and *P. flexilis*); Enantiomeric Distribution of Terpenoids in *Pinus* Species

**DOI:** 10.3390/molecules27175658

**Published:** 2022-09-02

**Authors:** Elizabeth Ankney, Kathy Swor, Prabodh Satyal, William N. Setzer

**Affiliations:** 1 Independent Researcher, 141 W. 17th St., Lafayette, OR 97127, USA; 2 Independent Researcher, 1432 W. Heartland Dr., Kuna, ID 83634, USA; 3 Aromatic Plant Research Center, 230 N 1200 E, Suite 100, Lehi, UT 84043, USA; 4 Department of Chemistry, University of Alabama in Huntsville, Huntsville, AL 35899, USA

**Keywords:** ponderosa pine, shore pine, limber pine, monoterpenoids, enantiomers, chiral GC-MS

## Abstract

*Pinus* species are important in traditional medicine throughout their ranges, and pine essential oils are of interest in aromatherapy and as topical treatments. In this work, the leaf (needle) essential oils of *Pinus ponderosa* var. *ponderosa* and *Pinus contorta* subsp. *contorta* from Oregon and *Pinus flexilis* growing in Idaho, have been obtained by hydrodistillation and analyzed by gas chromatographic techniques. The leaf essential oil of *P. ponderosa* was dominated by β-pinene (21.5–55.3%), methyl chavicol (8.5–41.5%), α-pinene (3.6–9.6%), δ-3-carene (3.6–6.2%), and α-terpineol (1.4–5.3%). The major components of *P. contorta* essential oil were β-phellandrene (23.8%), terpinen-4-ol (11.0%). The essential oil of *P. flexilis* was dominated by α-pinene (37.1%), β-pinene (21.9%), bornyl acetate (12.8%), and camphene (8.5%). Chiral gas chromatography revealed the enantiomeric ratios of α-pinene and limonene to be variable, but (−)-β-pinene predominated in *Pinus* essential oils.

## 1. Introduction

Numerous members of the genus *Pinus* (Pinaceae) are used in traditional medicine in their native ranges [1] and several essential oils derived from the genus are commercially important for use in aromatherapy and topical therapy applications, such as Scots pine (*Pinus sylvestris* L.), black pine (*Pinus nigra* J.F. Arnold), jack pine (*Pinus bansksiana* Lamb.), and white pine (*Pinus strobus* L.) [2]. In this work, the leaf essential oils of *Pinus ponderosa* Douglas ex C. Lawson var. *ponderosa*, *Pinus contorta* Douglas ex Loudon subsp. *contorta*, and *Pinus flexilis* E. James have been investigated for their chemical compositions and terpenoid enantiomeric distributions. In the case where essential oils are used therapeutically (e.g., aromatherapy) the different compositions and enantiomers may have very different biological activities. For commercial essential oils, the chemical compositions and enantiomeric distribution can be valuable for assessing the quality and consistency of the essential oil as well as a potential screen for adulteration or contamination.

**Figure 1:**
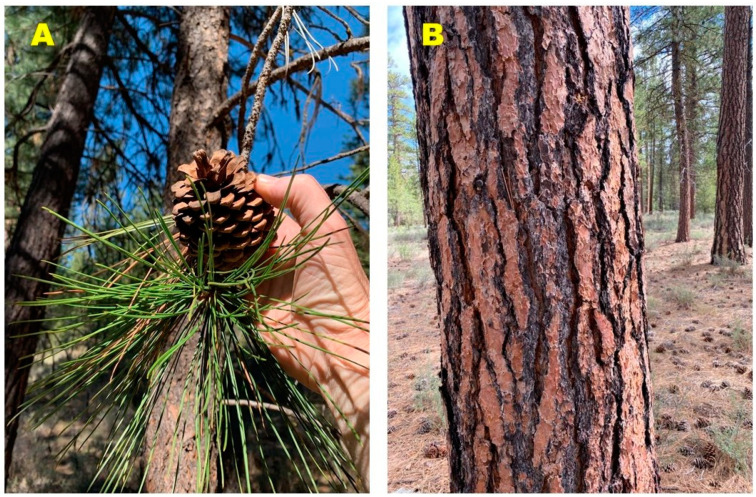
*Pinus ponderosa* var. *ponderosa* from central Oregon. (**A**) Leaves (needles) and cone. (**B**) bark.

*Pinus ponderosa*, the ponderosa pine (Figure 1), is the most widespread species of pine in western North America and ranges from British Columbia, south through the Cascade Range, the Sierra Nevada range of California, the Rocky Mountains and into the southwestern mountains of Utah, Arizona, and New Mexico. World Flora Online currently lists 11 subtaxa for the species [3], but the taxonomy is not resolved [4]. However, two varieties of the species are generally recognized: *Pinus ponderosa* var. *ponderosa*, the Pacific ponderosa pine, which ranges from southern British Columbia, south through the mountains of Washington, Oregon, and California, and *Pinus ponderosa* var. *scopulorum* Engelm., the Rocky Mountain ponderosa pine, found in eastern Montana, western North and South Dakota and Nebraska, Wyoming, Nebraska, northern and central Colorado and Utah [5]. Flathead Native Americans used the boughs of *P. ponderosa* in sweat lodges to treat muscular pains, while the Navajo people took a decoction of the needles for coughs and fever [6].

**Figure 2:**
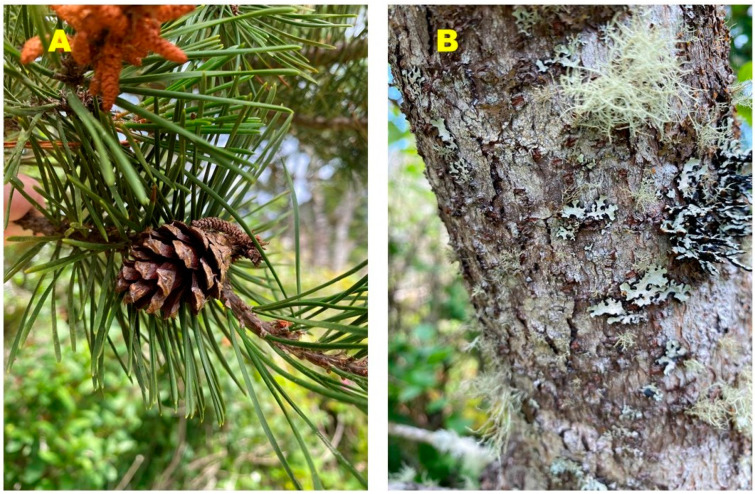
*Pinus contorta* subsp. *contorta* from the central Oregon coast. (**A**) Leaves (needles) and cone. (**B**) bark.

The native range of *P. contorta* is western North America, where there are three recognized subspecies: *P. contorta* subsp. *latifolia* (Engelm.) Critchf., the Rocky Mountain lodgepole pine, is found in the Rocky Mountains from the Yukon, south through Colorado; *P. contorta* subsp. *murrayana* (Balf.) Engelm., the Sierra lodgepole pine, found along the Cascade Range from Washington, through Oregon, and into northern California, and the Sierra Nevada Range in California; and *P. contorta* subsp. *contorta*, the shore pine (Figure 2), which ranges along the Pacific coast from southern Alaska, south to northwestern California [7,8]. The Haisla and Hanaksiala Native Americans used smoldering twigs of *P. contorta* subsp. *contorta* to alleviate pain and swelling of arthritic or injured joints [6].

**Figure 3:**
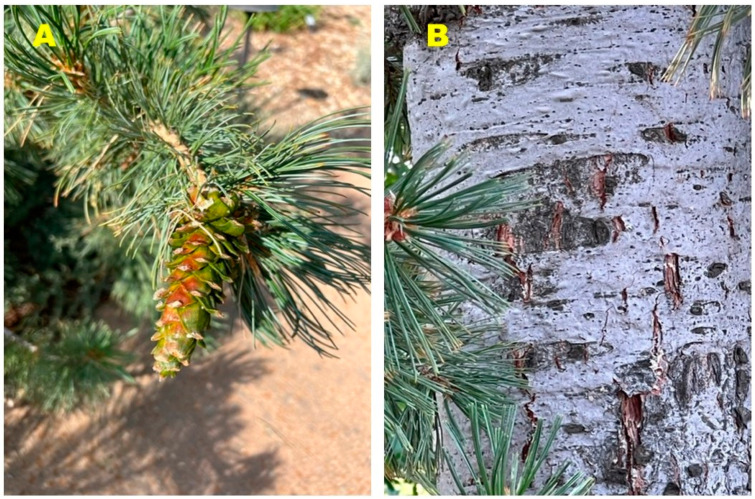
*Pinus flexilis* from southwestern Idaho. (**A**) Leaves (needles) and cone. (**B**) bark.

*Pinus flexilis* (Figure 3) naturally ranges in the Rocky Mountains of western North America, from southwest Alberta and southeast British Columbia, south through Colorado and New Mexico. It is also found in the mountains of Utah, Idaho, Nevada, and California [9]. The Navajo people used *P. flexilis* as cough medicine and to reduce fever [6]. As part of our investigation into the essential oil compositions of *Pinus* species [10,11], we have examined the compositions of the leaf essential oils of *P. ponderosa* var. *ponderosa* from La Pine, Oregon, *P. contorta* subsp. *contorta* from Ona Beach, Oregon, and *Pinus flexilis* from Boise, Idaho. As far as we are aware, this is the first report on the leaf oil composition of *P. flexilis* and the first report on the enantiomeric distributions of terpenoids in these *Pinus* species.

## 2. Results and Discussion

### 2.1. Chemical Composition of Pinus ponderosa var. ponderosa

Hydrodistillation of three samples of fresh leaves of *P. ponderosa* var. *ponderosa* gave colorless essential oils in 0.321%, 0.399%, and 0.463% (*w/w*) yield, which are comparable to those obtained in previous studies (0.1–0.6%) [12,13,14]. The essential oil compositions are presented in Table 1. A total of 118 compounds were identified in the essential oils accounting for >99% of the composition. The major components in the essential oils were β-pinene (21.5–55.3%), methyl chavicol (8.5–41.5%), α-pinene (3.6–9.6%), δ-3-carene (3.6–6.2%), and α-terpineol (1.4–5.3%).

There have been several investigations into the essential oil composition of *P. ponderosa* from different geographical locations, including California (USA) [13,14], British Columbia (Canada) [19], Washington (USA) [20], Poland [21], and Arizona (USA) [22]. Although there is much variation in the concentrations, the major components of *P. ponderosa* leaf essential oils reported in the literature have been α-pinene (10.2–69.3%), β-pinene (2.1–66.0%), myrcene (1.4–7.4%), δ-3-carene (up to 41.8%), α-terpineol (up to 7.5%) and methyl chavicol (1.8–20.4%). Thus, the essential oil compositions of Oregon *P. ponderosa*, subsp. *ponderosa* in this work are qualitatively similar to previous reports for *P. ponderosa*, and the wide chemical variations are likely due to geographical locations and/or genetic differences.

### 2.2. Chemical Composition of Pinus contorta Subsp. contorta

The fresh leaves of *P. contorta* subsp. *contorta* were hydrodistilled to give a colorless essential oil in 0.674% (*w/w*) yield. A previous report by Adams and co-workers indicated an essential oil yield of only 0.1% [23]. The essential oil composition is summarized in Table 2. A total of 55 compounds were identified accounting for 98.2% of the essential oil composition. The dominant components in the essential oil were the monoterpenoids β-phellandrene (23.8%), terpinen-4-ol (11.0%), thymol (6.6%), and chavicol (5.3%). Adams and co-workers have reported the leaf essential oils of *P. contorta* subsp. *contorta*, *P. contorta* subsp. *latifolia*, and *P. contorta* subsp. *murrayana* [23]. There are some notable differences between the leaf essential oil composition of the Oregon sample (this work) and those from coastal Washington [23]. The β-phellandrene concentration was lower than the Washington samples (39.2–61.5%), but γ-terpinene and terpinen-4-ol concentrations were higher than the Washington samples (0.6–1.7% and 0.3%, respectively), and neither chavicol nor thymol were detected in the Washington samples.

β-Phellandrene also dominated the essential oils of *P. contorta* subsp. *latifolia* from Alberta, Canada (34.3% β-phellandrene) [24] and *P. contorta* subsp. *murrayana* (37.2% β-phellandrene) [11]. In contrast, however, the concentration of terpinen-4-ol was relatively minor in both *P. contorta* subsp. *latifolia* (0.5%) and *P. contorta* subsp. *murrayana* (1.9%). Thymol was a minor component (0.3%) in *P. contorta* subsp. *murrayana*, and not observed in *P. contorta* subsp. *latifolia*. Chavicol was not observed either the *latifolia* or *murrayana* subspecies. Conversely, β-pinene was an abundant constituent of *P. contorta* subsp. *latifolia* (30.5%) and *P. contorta* subsp. *murrayana* (17.0%) as was α-terpineol (4.3% and 11.6%, respectively).

### 2.3. Chemical Composition of Pinus flexilis

Hydrodistillation of the fresh leaves (needles) of *P. flexilis* gave a colorless essential oil in 0.273% (*w/w*) yield. There have been no previous reports on *P. flexilis* essential oil yields. However, essential oils from *Pinus* species have been obtained in yields ranging from 0.08% (*P. rigida*) to 2.33% (*P. pumila*) [14]. The essential oil composition is presented in Table 3. A total of 102 compounds were identified in the leaf essential oil of *P. flexilis*, accounting for 99.7% of the composition. The major components in the essential oil were α-pinene (37.1%), β-pinene (21.9%), bornyl acetate (12.8%), and camphene (8.5%).

### 2.4. Enantiomeric Distribution of Terpenoids

The enantiomeric distributions of several terpenoid essential oil components have been determined by chiral gas chromatography-mass spectrometry. The enantiomeric distributions of terpenoid components of *P. ponderosa* var. *ponderosa*, *P. contorta* subsp. *contorta*, and *P. flexilis* essential oils are summarized in Table 4.

In *P. ponderosa* var. *ponderosa* essential oil, the (−)-enantiomer was the dominant stereoisomer in all monoterpenoids assessed. In the case of limonene and terpinen-4-ol, the (−)-enantiomer was only is slight excess over the (+)-enantiomer, however. In the case of *P. contorta* subsp. *contorta*, the (−)-enantiomer was dominant in α-pinene, β-pinene, α-phellandrene, limonene, β-phellandrene, borneol, and α-terpineol, which is comparable to the distribution found in *P. contorta* subsp. *murrayana* [11] as well as *P. ponderosa* var. *ponderosa* (above). Interestingly, the enantiomeric distribution for terpinen-4-ol was (+)53.0:(−)47.0 in *P. c.* subsp. *contorta*, but reversed in *P. c.* subsp. *murrayana*, (+)39.9:(−)60.1. In *P. flexilis*, the (−)-enantiomers dominated in α-pinene, camphene, β-pinene, α-phellandrene, β-phellandrene, and α-terpineol, while the (+)-enantiomers were exclusively observed for sabinene, fenchone, and β-bisabolene. As observed in *P. ponderosa* var. *ponderosa*, the (−)-enantiomers were slightly higher than the (+)-enantiomers for limonene and for terpinen-4-ol in *P. flexilis*.

The enantiomeric distributions for α-pinene, β-pinene, and limonene have been assessed for several *Pinus* species, which are listed in Table 5 for comparison. A perusal of Table 5 reveals that the enantiomeric distribution of α-pinene and limonene in *Pinus* species is variable both between species and within species. Allenspach and co-workers found that (+)-α-pinene generally predominated in primary essential oils of *P. sylvestris* and *P. cembra*, but that *P. mugo* and *P. nigra* were generally dominated by (−)-α-pinene [25]. The enantiomeric distribution in β-pinene in *Pinus* species, however, is consistently dominated by (−)-β-pinene.

The dominant enantiomer for α-pinene and β-pinene in *P. flexilis* were the (−)-enantiomers. α-Pinene enantiomeric distributions are generally variable in *Pinus* species, but (−)-β-pinene generally predominates in the genus (see above). The enantiomeric distribution of limonene also seems to be variable in *Pinus* species (see above), but (−)-limonene was the major enantiomer in *P. flexilis*. (−)-Camphene was the dominant enantiomer in *P. flexilis*, which was also found to be the case for *Pinus uncinata* subsp. *uliginosa* (G.E.Neumann ex Wimm.) Businský, *Pinus uncinata* Ramond ex DC., *Pinus peuce* Griseb., *Pinus mugo* Turra, *Pinus nigra* J.F. Arnold, *Pinus pinaster* Aiton, and *Pinus cembra* L. [26]. Interestingly, the enantiomeric distribution for camphene in *Pinus sylvestris* L. is variable depending on geographical source; (−)-camphene dominated in *P. sylvestris* from Poland and from Korea, whereas (+)-camphene dominated the essential oils from Austria and Italy [26]. (−)-Borneol and (−)-bornyl acetate were the exclusive enantiomers in *P. flexilis* essential oil, which was also observed in *P. contorta* subsp. *latifolia* (Engelm.) Critchf. [11].

## 3. Materials and Methods

### 3.1. Plant Material

Fresh plant material of *P. ponderosa* was collected from three individual mature trees growing near La Pine, Oregon (#1, 43°46′28″ N, 121°32′33″ W, elev. 1288 m; #2, 43°46′24″ N, 121°32′30″ W, elev. 1283 m; #3, 43°45′51″ N, 121°31′47″ W, elev. 1294 m), on 18 May 2021. *Pinus contorta* subsp. *contorta* was collected from a mature tree near Ona Beach, Oregon (44°31′16″ N, 124°4′13″ W, 3.0 m elevation) on 6 July 2021. The trees were identified in the field by E. Ankney using the field guide by Turner and Kuhlmann [30] and confirmed by comparison with samples from the C.V. Starr Virtual Herbarium, New York Botanical Garden (http://sweetgum.nybg.org/science/vh/, accessed on 14 January 2022). Leaves of *P. flexilis* were collected from a mature tree growing on the grounds of the Idaho Botanical Garden (43°36′04″ N, 116°09′35″ W, 862 m elevation) on 29 July 2021. The tree was identified by Daniel Murphy, Collections Curator of the Idaho Botanical Garden. Voucher specimens have been deposited in the University of Alabama in Huntsville herbarium. The fresh leaves (needles) of each tree sample were hydrodistilled for 3 h using a Likens-Nickerson apparatus to give colorless essential oils (Table 6). The essential oils were stored under refrigeration (−20 °C) until analysis. Commercial *Pinus* essential oil samples from the collection from the Aromatic Plant Research Center (APRC) were analyzed as received.

### 3.2. Gas Chromatography–Mass Spectrometry

Gas chromatographic–mass spectral (GC-MS) analysis of the *Pinus* essential oils was carried as previously described [31]: Shimadzu GCMS-QP2010 Ultra, ZB-5ms fused silica capillary column (60 m length, 0.25 mm diameter, 0.25 μm film thickness), He carrier gas, 2.0 mL/min flow rate, injection and ion source temperatures 260 °C; GC oven program 50 °C to 260 °C at 2.0 °C/min; 0.1 μL of a 5% (*w*/*v*) sample of essential oil in CH_2_Cl_2_ injected, split mode, 24.5:1 split ratio. Retention index (RI) values were calculated using a linear equation by Van den Dool and Kratz [32]. Identification of the essential oil components was carried out by comparison of MS fragmentation and comparison of retention indices (RI) with those available in the databases [15,16,17,18]. Representative gas chromatograms of the *Pinus* species are shown in supplementary Figure S1.

### 3.3. Gas Chromatography–Flame Ionization Detection

The GC-FID analysis was carried out as previously described [33]: Shimadzu GC 2010 equipped with flame ionization detector, a split/splitless injector, and Shimadzu autosampler AOC-20i; ZB-5 capillary column (60 m × 0.25 mm i.d.; film thickness 0.25 μm); He carrier gas, 1.0 mL/min flow rate; GC oven program as above for GC-MS; injector and detector temperatures maintained at 260 °C; 0.1 μL of a 5% (*w/v*) solution in CH_2_Cl_2_ injected, split mode, 31:1 split ratio. The percent compositions of the essential oil components were determined from peak areas and standardized using external standards of representative compounds from each compound class (α-pinene, β-pinene, camphene, limonene, menthol, borneol, (*E*)-β-caryophyllene, eugenol, and methyl chavicol).

### 3.4. Chiral Gas Chromatography–Mass Spectrometry

Chiral GC-MS of the leaf essential oils was carried out, as reported previously [34]: Shimadzu GCMS-QP2010S, electron impact (EI) mode, electron energy = 70 eV; scan range = 40–400 amu, scan rate = 3.0 scans/s; Restek B-Dex 325 chiral capillary GC column (30 m length × 0.25 mm inside diameter × 0.25 μm film thickness). Oven temperature program: starting temperature = 50 °C, temperature increased 1.5 °C/min to 120 °C, then 2 °C/min to 200 °C, and kept at 200 °C for an additional 5 min; carrier gas was helium, flow rate = 1.8 mL/min. For each essential oil sample, a 3% *w/v* solution in CH_2_Cl_2_ was prepared, and 0.1 μL was injected using a split ratio of 1:45. The enantiomers of the monoterpenoids were identified by comparison of retention times with authentic samples obtained from Sigma-Aldrich (Milwaukee, WI, USA). The enantiomer percentages were determined from peak areas.

## 4. Conclusions

The leaf essential oil compositions of *P. ponderosa* var. *ponderosa* and *P. contorta* subsp. *contorta* from Oregon, USA, have been determined. The enantiomeric distributions of these two *Pinus* species are reported for the first time. The chemical composition as well as the enantiomeric distribution for *P. flexilis* from Idaho, USA, are reported for the first time. Both α-pinene and limonene show considerable variation in enantiomeric distribution between and within *Pinus* species, but (−)-β-pinene is consistently the more dominant enantiomer. This work adds to our knowledge of the essential oil compositions of the genus *Pinus*. Additional studies on chemical compositions as well as enantiomeric distributions of members of the Pinaceae are underway in our laboratories.

## Figures and Tables

**Table 1:** Chemical composition of *Pinus ponderosa* var. *ponderosa* leaf essential oil.

RI_calc_	RI_db_	Compound	% Composition		
			Tree #1	Tree #2	Tree #3
919	919	Hashishene	tr	---	---
922	923	Tricyclene	tr	tr	tr
925	926	α-Thujene	tr	tr	tr
932	932	α-Pinene	3.6	5.7	9.6
946	948	α-Fenchene	tr	tr	tr
948	950	Camphene	0.1	0.2	0.3
970	970	3,7,7-Trimethyl-1,3,5-cycloheptatriene	tr	tr	tr
971	971	Sabinene	0.1	0.1	0.1
978	978	β-Pinene	21.5	35.3	55.3
988	989	Myrcene	1.7	1.3	1.7
999	1000	δ-2-Carene	tr	---	---
1006	1006	α-Phellandrene	tr	tr	tr
1009	1008	δ-3-Carene	3.6	5.5	6.2
1015	1015	1,4-Cineole	tr	tr	tr
1016	1017	α-Terpinene	tr	0.1	0.1
1019	1022	*m*-Cymene	tr	tr	tr
1024	1025	*p*-Cymene	0.1	0.1	0.1
1028	1030	Limonene	0.8	1.1	1.3
1030	1031	β-Phellandrene	0.9	1.3	1.7
1034	1034	(*Z*)-β-Ocimene	0.7	0.7	tr
1045	1045	(*E*)-β-Ocimene	0.1	tr	tr
1057	1057	γ-Terpinene	0.1	0.1	0.1
1070	1069	*cis*-Linalool oxide (furanoid)	tr	tr	tr
1080	1082	*p*-Mentha-2,4(8)-diene	tr	tr	tr
1084	1086	Terpinolene	0.4	0.7	0.8
1086	1086	*trans*-Linalool oxide (furanoid)	0.1	---	0.1
1089	1091	*p*-Cymenene	tr	---	tr
1090	1090	2-Nonanone	---	0.1	tr
1099	1101	Linalool	2.1	0.4	0.3
1104	1104	Nonanal	0.1	tr	tr
1118	1119	*endo*-Fenchol	0.1	tr	tr
1124	1124	*cis-p*-Menth-2-en-1-ol	tr	tr	tr
1126	1126	α-Campholenal	tr	tr	tr
1127	1127	*allo*-Ocimene	---	tr	---
1137	1137	Nopinone	0.2	tr	0.1
1140	1140	*trans*-Pinocarveol	0.4	0.1	tr
1142	1142	*trans-p*-Menth-2-en-1-ol	---	0.1	---
1145	1145	Camphor	---	tr	---
1154	1156	Camphene hydrate	0.1	0.1	0.1
1155	1155	Hexyl isobutyrate	---	---	tr
1160	1160	*trans*-Pinocamphone	0.2	0.2	0.3
1161	1164	Pinocarvone	0.3	0.1	0.1
1170	1170	(2*E*)-Nonen-1-ol	0.1	tr	0.1
1171	1171	*p*-Mentha-1,5-dien-8-ol	0.1	tr	tr
1175	1176	*cis*-Pinocamphone	0.2	0.2	0.2
1180	1180	Terpinen-4-ol	0.4	0.3	0.2
1187	1186	*p*-Cymen-8-ol	0.3	0.1	---
1196	1195	α-Terpineol	5.3	1.4	3.0
1199	1197	Methyl chavicol (= Estragole)	41.5	27.4	8.5
1206	1206	Decanal	---	0.1	0.1
1208	1208	Verbenone	tr	tr	---
1228	1229	Thymol methyl ether	---	---	tr
1252	1253	(*Z*)-Anethole	---	tr	---
1253	1254	Piperitone	---	tr	---
1278	1276	(2*E*)-Decen-1-ol	---	0.1	---
1283	1282	Bornyl acetate	0.2	0.1	0.1
1285	1285	(*E*)-Anethole	2.3	1.6	0.1
1292	1293	2-Undecanone	---	0.1	---
1313	1314	Carvenolide	0.1	---	---
1322	1322	Myrtenyl acetate	0.1	0.1	tr
1345	1346	α-Terpinyl acetate	0.3	0.3	0.2
1372	1370	(2*E*)-Undecen-1-ol	0.7	0.3	0.3
1375	1375	α-Copaene	0.1	0.2	0.2
1383	1382	β-Bourbonene	---	tr	---
1387	1387	β-Cubebene	tr	0.1	tr
1389	1390	*trans*-β-Elemene	0.1	---	---
1389	1389	(5*Z*)-Decen-1-yl acetate	---	0.5	0.4
1399	1403	Methyl eugenol	0.1	tr	---
1409	1410	Dodecanal	0.1	0.1	0.1
1419	1417	(*E*)-β-Caryophyllene	0.5	0.5	0.1
1429	1430	β-Copaene	tr	tr	tr
1432	1432	*trans*-α-Bergamotene	0.5	0.1	0.1
1438	1438	Aromadendrene	0.3	tr	0.2
1442	1442	Guaia-6,9-diene	---	---	tr
1447	1447	Geranyl acetone	---	tr	---
1448	1448	*cis*-Muurola-3,5-diene	---	tr	tr
1452	1452	(*E*)-β-Farnesene	0.1	tr	tr
1455	1454	α-Humulene	0.1	0.1	tr
1459	1457	*allo*-Aromadendrene	---	---	tr
1461	1463	*cis*-Muurola-4(14),5-diene	0.1	0.1	tr
1467	1469	Ethyl (*E*)-cinnamate	0.2	---	0.1
1469	1470	(2*E*)-Undecenyl acetate	0.1	0.3	tr
1471	1472	*trans*-Cadina-1(6),4-diene	tr	0.1	0.1
1474	1475	γ-Muurolene	0.2	0.4	0.2
1480	1480	Germacrene D	0.4	0.9	0.3
1488	1489	β-Selinene	0.4	0.1	0.2
1491	1492	*trans*-Muurola-4(14),5-diene	0.1	0.1	0.1
1495	1495	2-Tridecanone	---	0.3	---
1496	1497	Bicyclogermacrene	0.8	---	0.5
1498	1497	α-Muurolene	0.3	0.5	0.3
1512	1512	γ-Cadinene	0.9	1.5	1.0
1518	1518	δ-Cadinene	1.6	2.8	1.9
1519	1519	*trans*-Calamenene	tr	tr	0.1
1522	1521	Zonarene	tr	tr	0.1
1532	1533	*trans*-Cadina-1,4-diene	tr	0.1	0.1
1536	1538	α-Cadinene	0.1	0.1	0.1
1540	1541	α-Calacorene	tr	tr	tr
1561	1561	(*E*)-Nerolidol	---	1.0	---
1561	1560	Dodecanoic acid	0.5	0.2	0.3
1574	1574	Germacrene D-4α-ol	---	0.6	---
1577	1576	Spathulenol	1.0	---	0.6
1581	1582	Caryophyllene oxide	0.2	0.1	tr
1586	1590	Globulol	0.1	0.1	0.1
1593	1598	Ethyl dodecanoate	0.1	---	---
1625	1624	Muurola-4,10(14)-dien-1β-ol	tr	0.1	tr
1627	1628	1-*epi*-Cubenol	tr	0.1	0.1
1642	1643	τ-Cadinol	0.3	0.5	0.3
1644	1644	τ-Muurolol	0.3	0.6	0.5
1655	1655	α-Cadinol	0.5	0.7	0.5
1664	1664	Brevifolin (= Xanthoxylin)	0.1	---	---
1675	1670	(6*Z*)-Pentadecen-2-one	0.1	0.2	---
1765	1769	Benzyl benzoate	---	0.1	tr
1794	1796	(9Z)-Hexadecenal	---	0.1	tr
1816	1817	Hexadecanal	tr	0.1	0.1
1866	1869	Benzyl salicylate	---	0.1	---
1991	1989	Manoyl oxide	0.2	0.1	0.1
1995	1997	9β-Isopimara7,15-diene	---	0.1	0.1
2290	2297	Methyl isopimarate	0.1	0.1	tr
		Monoterpene hydrocarbons	33.6	52.3	77.3
		Oxygenated monoterpenoids	10.5	3.4	4.5
		Sesquiterpene hydrocarbons	6.7	7.7	5.5
		Oxygenated sesquiterpenoids	2.4	3.7	2.0
		Diterpenoids	0.3	0.3	0.1
		Benzenoid aromatics	44.2	29.2	8.7
		Others	1.7	2.5	1.4
		Total identified	99.5	99.1	99.5

RI_calc_ = Retention indices calculated in reference to a homologous series of *n*-alkanes on a ZB-5ms column. RI_db_ = Retention indices obtained from the databases [15,16,17,18]. tr = “trace” (<0.05%). --- = not detected.

**Table 2:** Chemical composition of *Pinus contorta* subsp. *contorta* leaf essential oil.

RI_calc_	RI_db_	Compound	% Composition
782	782	Prenol	1.1
801	801	Hexanal	0.6
848	849	(2*E*)-Hexenal	0.5
851	853	(3*Z*)-Hexenol	0.3
923	923	Tricyclene	0.1
925	927	α-Thujene	0.2
933	932	α-Pinene	1.2
949	950	Camphene	0.2
959	959	Benzaldehyde	2.0
972	971	Sabinene	0.2
977	978	β-Pinene	0.5
989	989	Myrcene	1.0
989	990	Dehydro-1,8-cineole	0.1
1007	1006	α-Phellandrene	0.6
1009	1008	δ-3-Carene	0.2
1014	1015	1,4-Cineole	3.7
1017	1017	α-Terpinene	3.6
1024	1024	*p*-Cymene	1.5
1029	1030	Limonene	2.0
1030	1031	β-Phellandrene	23.8
1035	1034	(*Z*)-β-Ocimene	1.1
1057	1057	γ-Terpinene	6.8
1070	1069	*cis*-Linalool oxide (furanoid)	0.2
1085	1086	Terpinolene	2.2
1086	1086	*trans*-Linalool oxide (furanoid)	0.4
1089	1091	*p*-Cymenene	0.3
1100	1099	Linalool	0.1
1124	1124	*cis-p*-Menth-2-en-1-ol	1.8
1135	1136	Terpin-3-en-1-ol	2.3
1142	1142	*trans-p*-Menth-2-en-1-ol	1.2
1146	1145	Camphor	0.6
1177	1179	2-Isopropenyl-5-methyl-4-hexenal	0.6
1180	1180	Terpinen-4-ol	11.0
1187	1186	*p*-Cymen-8-ol	1.7
1187	1188	*trans*-β-Ocimenol	0.3
1195	1195	α-Terpineol	2.4
1196	1197	Estragole (= Methyl chavicol)	0.4
1199	1200	γ-Terpineol	0.9
1237	1237	Pulegone	0.4
1249	1250	Chavicol	5.3
1277	1277	Phellandral	0.3
1286	1285	(*E*)-Anethole	0.3
1289	1289	Thymol	6.6
1353	1356	Eugenol	0.3
1444	1442	Guaia-6,9-diene	0.8
1483	1480	Germacrene D	0.2
1564	1560	Dodecanoic acid	1.7
1573	1571	(3*Z*)-Hexenyl benzoate	1.6
1579	1576	Spathulenol	0.5
1627	1627	Benzophenone	0.2
1766	1769	Benzyl benzoate	0.5
1868	1869	Benzyl salicylate	0.5
1960	1958	Palmitic acid	0.6
2012	2016	Juvabione	0.6
2052	2053	Manool	0.4
		Monoterpene hydrocarbons	45.3
		Oxygenated monoterpenoids	34.2
		Sesquiterpene hydrocarbons	0.9
		Oxygenated sesquiterpenoids	0.5
		Diterpenoids	0.4
		Benzenoid aromatics	11.5
		Others	5.5
		Total identified	98.2

RI_calc_ = Retention indices calculated in reference to a homologous series of *n*-alkanes on a ZB-5ms column. RI_db_ = Retention indices obtained from the databases [15,16,17,18].

**Table 3:** Chemical composition of *Pinus flexilis* leaf essential oil.

RI_calc_	RI_db_	Compound	% Composition
801	801	Hexanal	0.2
848	849	(2*E*)-Hexenal	0.7
850	853	(3*Z*)-Hexenol	0.2
863	867	1-Hexanol	0.1
880	880	Santene	0.1
900	900	Nonane	tr
923	923	Tricyclene	0.7
925	925	α-Thujene	tr
933	933	α-Pinene	37.1
951	953	Camphene	8.5
953	953	Thuja-2,4(10)-diene	tr
972	972	Sabinene	0.3
979	978	β-Pinene	21.9
989	989	Myrcene	1.5
1007	1007	α-Phellandrene	0.1
1017	1017	α-Terpinene	0.1
1024	1024	*p*-Cymene	0.1
1030	1030	Limonene	3.3
1031	1031	β-Phellandrene	2.2
1034	1034	(*Z*)-β-Ocimene	0.1
1045	1045	(*E*)-β-Ocimene	tr
1057	1057	γ-Terpinene	0.2
1085	1086	Terpinolene	1.0
1088	1090	Fenchone	0.1
1089	1093	*p*-Cymenene	tr
1096	1099	6-Camphenone	0.1
1100	1100	Undecane	0.4
1104	1104	Nonanal	tr
1119	1120	*endo*-Fenchol	tr
1124	1124	*cis-p*-Menth-2-en-1-ol	tr
1126	1126	α-Campholenal	0.2
1138	1139	Nopinone	tr
1140	1141	*trans*-Pinocarveol	0.2
1142	1142	*trans-p*-Menth-2-en-1-ol	tr
1145	1145	*trans*-Verbenol	0.1
1147	1145	Camphor	0.1
1150	1150	α-Phellandren-8-ol	0.1
1155	1156	Camphene hydrate	0.1
1160	1160	*trans*-Pinocamphone	tr
1162	1164	Pinocarvone	tr
1171	1171	*p*-Mentha-1,5-dien-8-ol	0.3
1171	1173	Borneol	0.2
1180	1180	Terpinen-4-ol	0.2
1186	1186	*p*-Cymen-8-ol	0.1
1195	1195	α-Terpineol	1.5
1206	1205	Verbenone	tr
1228	1229	Thymyl methyl ether	0.2
1286	1287	Bornyl acetate	12.8
1291	1293	2-Undecanone	0.3
1294	1294	*trans*-Pinocarvyl acetate	tr
1300	1300	Tridecane	tr
1357	1357	2-Methylundecanal	0.1
1376	1375	α-Copaene	0.1
1409	1410	Dodecanal	0.1
1410	1408	Acora-3,7(14)-diene	tr
1420	1417	(*E*)-β-Caryophyllene	0.2
1430	1430	β-Copaene	tr
1452	1152	(*E*)-β-Farnesene	0.2
1455	1154	α-Humulene	tr
1475	1175	γ-Muurolene	tr
1481	1480	Germacrene D	0.2
1494	1494	2-Tridecanone	0.3
1498	1497	α-Muurolene	0.2
1507	1508	β-Bisabolene	0.6
1512	1512	γ-Cadinene	0.1
1518	1518	δ-Cadinene	0.3
1548	1549	α-Elemol	tr
1560	1560	(*E*)-Nerolidol	tr
1576	1576	Spathulenol	0.1
1627	1628	1-*epi*-Cubenol	tr
1641	1640	τ-Cadinol	0.1
1643	1644	τ-Muurolol	0.1
1647	1651	α-Muurolol (= δ-Cadinol)	tr
1655	1655	α-Cadinol	0.2
1664	1665	Intermedeol	tr
1668	1667	(6*Z*)-Pentadecen-2-one	tr
1684	1683	*epi*-α-Bisabolol	tr
1687	1688	α-Bisabolol	0.7
1696	1697	2-Pentadecanone	0.1
1707	1706	(2*E*,6*Z*)-Farnesal	tr
1717	1714	(2*E*,6*Z*)-Farnesol	0.1
1734	1737	(2*E*,6*E*)-Farnesal	tr
1782	1779	Dodecyl butyrate	tr
1815	1817	Hexadecanal	tr
1830	1832	Farnesyl acetate	tr
1964	1968	Sandaracopimara-8(14),15-diene	0.1
1993	1994	Manoyl oxide	0.3
1997	2000	9β-Isopimara-7,15-diene	0.1
2013	2007	18-Norabieta-8,11,13-triene	0.1
2085	2086	Abietadiene	tr
2145	2147	Abienol	tr
2182	2180	Sandaracopimarinal	0.1
2222	2231	Isopimarinal	0.2
2230	2236	Palustrinal	0.2
2234	---	Levopimarinal ^a^	tr
2241	2238	Methyl pimarate	tr
2262	2267	Dehydroabietal	tr
2292	2297	Methyl isopimarate	tr
2296	2302	Methyl levopimarate	tr
2307	2312	Abietal	tr
2330	2341	Methyl dehydroabietate	tr
2365	2366	Neoabietic acid	tr
		Monoterpene hydrocarbons	77.3
		Oxygenated monoterpenoids	16.0
		Sesquiterpene hydrocarbons	1.9
		Oxygenated sesquiterpenoids	1.2
		Diterpenoids	0.9
		Fatty acid derivatives	2.3
		Total identified	99.7

RI_calc_ = Retention index calculated with respect to a homologous series of *n*-alkanes on a ZB-5ms column. RI_db_ = Reference retention index obtained from the databases [15,16,17,18]. tr = trace (<0.05%). ^a^ Identification tentative; the MS is a good match (93% similarity match), but there is no reference RI available.

**Table 4:** Enantiomeric distribution of terpenoids of *Pinus ponderosa* var. *ponderosa*, *Pinus contorta* subsp. *contorta*, and *Pinus flexilis* leaf essential oils.

Terpenoid Compound	Enantiomeric Distribution, (+):(−)				
	*P. ponderosa*			*P. contorta*	*P. flexilis*
	Tree #1	Tree #2	Tree #3		
α-Pinene	53.3:46.7	20.3:79.7	6.2:93.8	27.5:72.5	4.8:95.2
Camphene	47.9:52.1	10.6:89.4	8.2:91.8	---	1.8:98.2
Sabinene	---	---	---	---	100:0
β-Pinene	1.9:98.1	1.7:98.3	1.7:98.3	0:100	3.2:96.8
α-Phellandrene	---	---	---	8.4:91.6	17.2:82.8
δ-3-Carene	72.1:27.9	0.7:99.3	1.0:99.0	---	---
Limonene	38.7:61.3	41.1:58.9	41.2:58.8	13.2:86.8	33.0:67.0
β-Phellandrene	2.3:97.7	0.9:99.1	1.3:98.7	0.6:99.4	3.5:96.5
Fenchone	---	---	---	---	100:0
Linalool	7.6:92.4	9.3:90.7	9.7:90.3	---	---
Camphor	---	---	---	0:100	---
Borneol	---	---	---	---	0:100
Terpinen-4-ol	37.2:62.8	30.7:69.3	39.3:60.7	53.0:47.0	43.5:56.5
α-Terpineol	2.6:97.4	3.6:96.4	2.8:97.2	35.5:64.5	8.8:91.2
Pulegone	---	---	---	100:0	---
Bornyl acetate	0:100	0:100	0:100	---	0:100
α-Terpinyl acetate	0:100	0:100	0:100	---	---
(*E*)-β-Caryophyllene	0:100	0:100	0:100	---	0:100
Germacrene D	0:100	0:100	0:100	---	0:100
β-Bisabolene	---	---	---	---	100:0
δ-Cadinene	0:100	0:100	0:100	---	0:100
(*E*)-Nerolidol	---	0.6:99.4	---	---	---

--- = not detected.

**Table 5:** Enantiomeric distribution, (+):(–), of monoterpene hydrocarbons in *Pinus* species leaf essential oils.

*Pinus* Species	Geographical Source	α-Pinene	β-Pinene	Limonene	Ref.
*Pinus banksiana* Lamb	Eastern Canada	74.5:25.5	3.0:97.0	8.4:91.6	APRC
	Eastern Canada	74.4:25.6	3.0:97.0	8.4:91.6	
*Pinus cembra* L.	Italy	64.4:35.6	0.8:99.2	0:100	[26]
*Pinus contorta* subsp. *murrayana* (Balf.) Engelm.	Oregon, USA	20.1:79.7	2.2:97.8	0:100	[11]
*Pinus contorta* Douglas ex Loudon subsp. *contorta*	Oregon, USA	27.5:72.5	0:100	13.2:86.8	This work
*Pinus flexilis* E. James	Idaho, USA	4.8:95.2	3.2:96.8	33.0:67.0	This work
*Pinus halepensis* Mill.	Portugal	59.1:40.9	4.7:95.3	---	[27]
*Pinus mugo* Turra (syn. *P. montana* Mill.)	Austria	49.2:50.8	0.9:99.1	28.1:71.9	[26]
	Italy	63.3:36.7	1.4:98.6	13.4:86.6	
	Korea	43.8:56.2	19.1:80.9	62.7:37.3	
*Pinus nigra* J.F. Arnold	Austria	16.9:83.1	6.7:93.3	23.8:76.2	[26]
	Albania	3.9:96.1	18.0:82.0	23.6:76.4	APRC
*Pinus peuce* Griseb.	Germany	26.8:73.2	3.7:96.3	29.2:70.8	[28]
	Germany	31.0:69.0	3.3:96.7	20.2:79.8	
*Pinus pinaster* Aiton	Italy	71.3:28.7	2.6:97.4	17.8:82.2	[26]
	Portugal	30.3:69.7	0.6:99.4	31.0:69.0	[27]
*Pinus pinea* L.	Portugal	48.3:51.7	0:100	0.4:99.6	[27]
*Pinus ponderosa* Douglas ex C. Lawson var. *ponderosa*	Oregon, USA	53.3:46.7	1.9:98.1	38.7:61.3	This work
	Oregon, USA	20.3:79.7	1.7:98.3	41.1:58.9	
	Oregon, USA	6.2:93.8	1.7:98.3	41.2:58.8	
*Pinus resinosa* Aiton	Eastern Canada	61.2:38.8	2.9:97.1	44.0:56.0	APRC
	Eastern Canada	63.0:37.0	2.5:97.5	38.8:61.2	
*Pinus strobus* L.	Eastern Canada	39.8:60.2	2.2:97.8	16.5:83.5	APRC
	Eastern Canada	40.2:59.8	2.4:97.6	16.5:83.5	
*Pinus sylvestris* L.	Poland	76.2:23.8	1.8:98.2	98.1:1.9	[26]
	Austria	23.2:76.8	3.5:96.5	25.9:74.1	
	Italy	13.5:86.5	3.6:96.4	29.3:70.7	
	Korea	33.4:66.6	4.9:95.1	66.7:33.3	
	Portugal	27.2:72.8	0.9:99.1	---	[27]
	Eastern Canada	67.1:32.9	2.3:97.7	21.8:78.2	APRC
	Eastern Canada	67.3:32.7	2.4:97.6	21.7:78.3	
*Pinus uncinata* subsp. *uliginosa* (G.E.Neumann ex Wimm.) Businský	Poland	65.6:34.4	11.7:88.3	63.6:36.4	[29]
*Pinus uncinata* Ramond ex DC.	Poland	58.4:41.6	9.1:90.9	11.7:88.3	[29]

APRC = Data from the commercial essential oil samples from the collection of the Aromatic Plant Research Center (Lehi, Utah, USA). --- = not detected.

**Table 6:** Collection and hydrodistillation details of *Pinus* species.

Tree Sample	Voucher Number	Mass Leaves	Mass Essential Oil
*Pinus ponderosa* var. *ponderosa* #1	EA-50553	33.25 g	106.6 mg
*Pinus ponderosa* var. *ponderosa* #2		33.39 g	133.2 mg
*Pinus ponderosa* var. *ponderosa* #3		67.72 g	313.8 mg
*Pinus contorta* subsp. *contorta*	EA-50554	15.82 g	106.7 mg
*Pinus flexilis*	KS-58231	115.62	315.7 mg

## Data Availability

All data are available in the manuscript.

## Supplementary Materials

The following supporting information can be downloaded at: https://www.mdpi.com/article/10.3390/molecules27175658/s1, Figure S1: Gas chromatograms of *Pinus ponderosa* var. *ponderosa* (**A**), *Pinus contorta* subsp. *contorta* (**B**), and *Pinus flexilis* (**C**).

## Sample Availability

Essential oil samples are no longer available.
