# Supplementary material for: Essential Oil Compositions of Pinus Species (P. contorta Subsp. contorta, P. ponderosa var. ponderosa, and P. flexilis); Enantiomeric Distribution of Terpenoids in Pinus Species
Source: Molecules. 2022 Sep 2;27(17):5658. doi: 10.3390/molecules27175658 (PMC9457545; doi:10.3390/molecules27175658)
Supplement: Supplementary file 1 [file molecules-27-05658-s001.zip › molecules-1900887-supplementary.pdf]

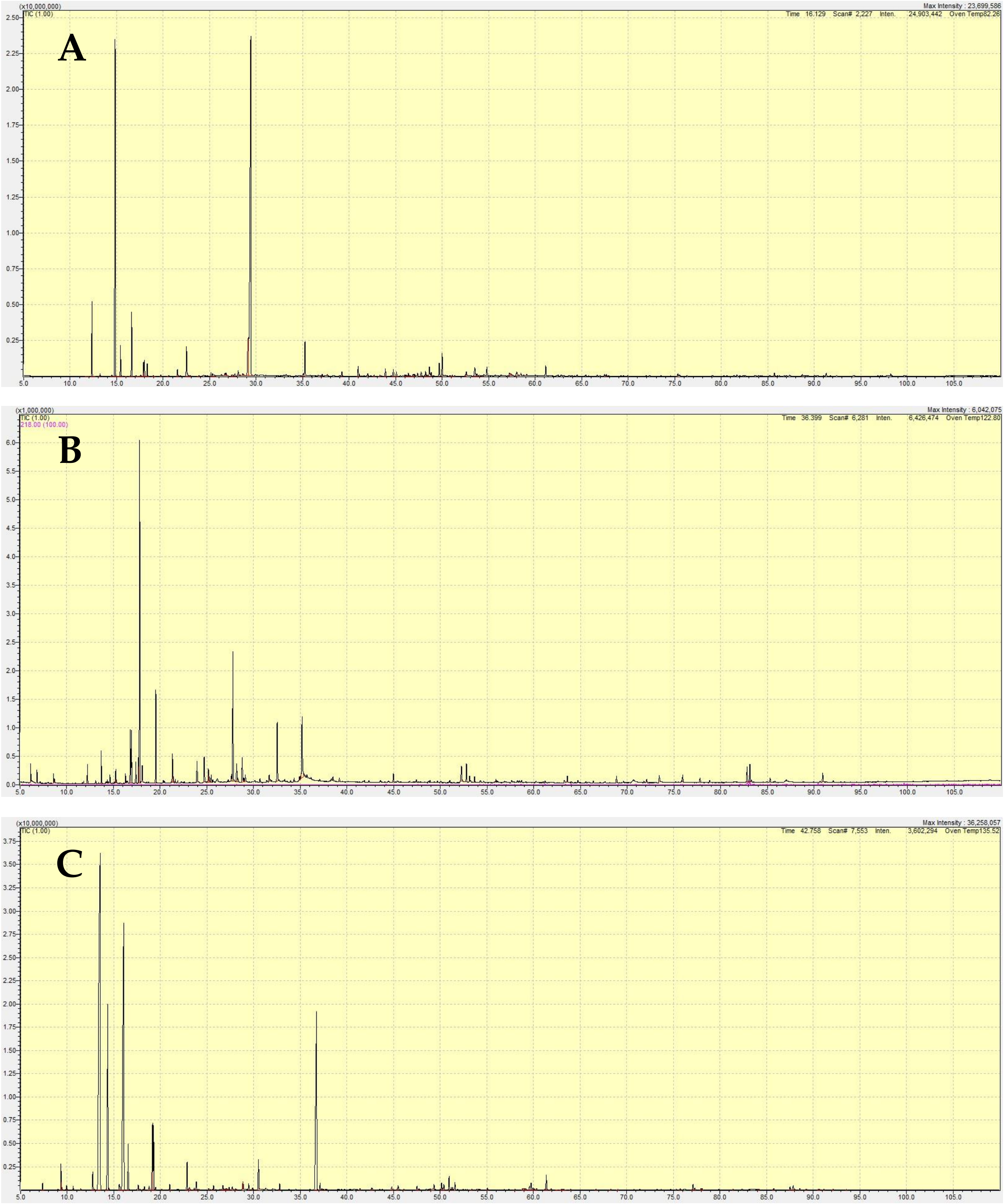

Figure S1. Gas chromatograms of *Pinus ponderosa* var. *ponderosa* (**A**), *Pinus contorta* subsp. *contorta* (**B**), and *Pinus flexilis* (**C**).
